# ^1^H NMR Spectroscopy Primitivo Red Wine Screening After Grape Pomace Repassage for Possible Toxin Contamination Removal

**DOI:** 10.3390/foods14050734

**Published:** 2025-02-21

**Authors:** Laura Del Coco, Chiara Roberta Girelli, Lucia Gambacorta, Michele Solfrizzo, Francesco Paolo Fanizzi

**Affiliations:** 1 Department of Biological and Environmental Science and Technology, University of Salento, Via Lecce-Monteroni, 73100 Lecce, Italychiara.girelli@unisalento.it (C.R.G.); 2 Institute of Sciences of Food Production (ISPA), National Research Council (CNR), Via Amendola 122/O, 70126 Bari, Italy; lucia.gambacorta@ispa.cnr.it (L.G.); michele.solfrizzo@ispa.cnr.it (M.S.)

**Keywords:** wine, Ochratoxin A, grape pomace repassage, ^1^H-NMR, metabolomics

## Abstract

Food safety and quality awareness have reached significant relevance as consumers are more interested in authentic foods and drinks with specific organoleptic values. Among foodstuffs, grape products can be contaminated by Ochratoxin A (OTA), a mycotoxin that can occur in red grape after infection with *Aspergillus carbonarius.* The high affinity of grape pomace with OTA makes its use advantageous as an adsorbing/decontaminating material whether the pomace is fresh, has undergone pressing, or has undergone a stabilizing process. The effects of different grape repassage treatments on wine metabolic profiles were studied by ^1^H NMR spectroscopy coupled with metabolomics. The relative quantification of discriminating metabolites for activated-carbon-treated samples revealed higher levels of ethyl acetate and succinate than for the grape-pomace-repassed wine samples. On the contrary, the latter exhibited a relatively high content of glycerol, lactate, tartaric, isobutanol, isopentanol, and polyphenols. Although a specific decrease in aromatic compounds such as gallic acid, tyrosine, and tyrosol was also observed compared with the controls, for the pomace-based processes, the activated carbon treatment led to a marked general impoverishment of the metabolomic profiles, with a reduction in organic acids and glycerol. The repassage of wine over the grape pomace did not significantly affect the quality attributes of the wine, offering an alternative natural adsorbing/decontaminating material for the removal of OTA.

## 1. Introduction

Among foodstuffs, grape products cover a wide group of foods such as grape berries and related processing derivatives, including wine, grape juice, distillates, vinegar, and jellies. All these products can be contaminated by harmful metabolites, in particular mycotoxin produced by specific fungi [1]. Among these, Ochratoxin A (OTA) is a naturally occurring secondary metabolite produced by different species of *Aspergillus* and *Penicillium* fungi genera [2]. Following cereals, wine is currently recognized as the second most significant source of OTA human exposure [1]. This mycotoxin has been officially classified as a potential human carcinogen (group 2B) by the International Agency for Research on Cancer (IARC) [3,4]. The molecular mechanisms of the oxidative pathway in OTA-mediated cytotoxicity (ROS accumulation, DNA damage, G1 arrest) in human immune cells have been recently described [5]. The OTA nephrotoxic, neurotoxic, teratogenic, and immunotoxic effects on human and animal health are also well described in the literature [4]. It was reported to have a correlation with the human Balkan endemic nephropathy (Ben), a chronic renal disease, and human urinary tract cancers [6]. After European Food Safety Authority (EFSA) OTA toxicity evaluations (CONTAM Panel) [7], the EU Commission (EC) introduced a maximum tolerable value of 2 µg/L in must and wine [8]. Being frequently present on the grapes surface, the skins are responsible for OTA contamination of wine [6]. The transfer of mycotoxin from grapes to the final products may occur at any step of the winemaking operation, from the berry crushing, when the toxin passes into the liquid, to the maceration process, in which the OTA level increases [6]. Thus, although the fermentation and clarification stages induce OTA reduction, the vinification process strictly affects the toxin content, thereby leading to the development of several OTA concentration reduction strategies into the final product [1,6,9,10]. Biological (yeast, bacteria, fungi, enzymes) and physical (heating, UV light) strategies are currently available to remove OTA from contaminated wines [1,6]. Biological methods use different microorganisms (yeast, bacteria, fungi) to degrade the mycotoxins in wine [1,6,11]. Physical methods include filtration, radiation, and thermal treatments [1,6]. Chemical methods involve the use of fining agents to reduce the OTA level in wine [12]. Among these, activated carbon showed a high affinity with OTA but the organoleptic properties and the quality parameters could be strongly affected [4,6,13]. Indeed, the efficacy of oenological charcoal in OTA removal was reported to be directly related to a polyphenol content decrease, with a resulting negative impact on wine quality [14,15]. Moreover, among the physical treatment proposed to reduce mycotoxins in wine, an innovative, environmentally friendly, and efficient decontamination procedure using grape pomace repassage was developed by Solfrizzo et al. [13]. Pomace treatment is not uncommon in wineries, since repassage over Recioto or Amarone pomace is largely used in the case of Venetian high-quality wines, like “Valpolicella Ripasso”, to give more body and sweetness aromas to Valpolicella red wines [13]. The repassage of contaminated wines over grape pomaces of the same variety showed a strong affinity for OTA, even after multiple uses (up to four times). Moreover, the mitigation effect using pomace repassage is a fast process and it can be observed after only 24 h after the repassage, reaching a 50–65% OTA reduction [13]. Recently, a winery prototype and a specific repassage process have been developed, patented, and used with excellent results (OTA removal of 70–80% from 1000 L of wine in just 5 h) [16]. Unlike other oenological fining agents, the high affinity of grape pomaces for OTA efficiently reduced its concentration in repassed wine without affecting its quality parameters such as color intensity and, above all, healthy compound content [13]. Although the role of grape pomace as a wine fining and clarification agent has been studied since 2013 [17], to the best of our knowledge, very few works have focused on the effect of repassage on wine quality parameters [18,19,20], and to date, no metabolomic studies are available. It should be noted that metabolomics coupled with NMR spectroscopy is widely used in food science since it is proven to be a reliable tool in the chemical characterization of food matrices for quality control, authenticity, and geographical origin assessment [21,22,23,24,25]. Specifically, NMR-based metabolomics is particularly suitable for wine screening analysis [26] and has been successfully applied to grape variety discrimination [27,28], PGI classification [29,30], adulteration detection [31], and wine authentication [32]. Thus, in this work, we characterized, for the first time, by a ^1^H-NMR- metabolomic method, the chemical profiles of Apulian high-quality Primitivo red wine [33], which is quite susceptible to OTA contamination [13], and was submitted to different grape pomace repassage. In particular, the ^1^H-NMR metabolic profiles of the untreated wine (control) samples were compared with activated carbon, fresh/stabilized Primitivo and fresh Aglianico grape pomace repassage treatments to investigate the possible variation in the wine metabolome and related quality parameters after the decontamination treatments.

## 2. Materials and Methods

### 2.1. Chemicals and Reagents

All chemical reagents for analysis were of analytical grade. Deuterium oxide (99.9 atom %D) containing 0.05% wt 3-(trimethylsilyl)propionic-2,2,3,3 d4 acid sodium salt (TSP) potassium phosphate monobasic was purchased from Armar Chemicals (Döttingen, Switzerland). Sodium azide was purchased by J.T. Baker (Phillipsburg, NJ, USA).

### 2.2. Wine Sampling

Primitivo is one of the most important and representative Apulian red grape varieties in Southern Italy, from which high-quality wine is produced, which is quite susceptible to OTA contamination [13,33]. In order to focus on possible metabolomic alteration due to decontamination procedures, minimizing any other variability source, Primitivo di Gioia cv OTA-free biological red wine samples from a 3000 L bulk tank representative of the commercial product were subjected to four different treatments (each treatment in triplicate or duplicate for a total of 11 different biological samples) and compared with untreated samples (3 controls). Therefore, in order to assess the differences produced in the metabolic profile of the same bulk product according to the specific performed treatment, the following samples were considered:

1. Controls, namely, untreated OTA-free Primitivo di Gioia red wine: three samples.
2. Repassage over Primitivo di Gioia fresh pomace: two different biological samples.
3. Repassage over Primitivo di Gioia stabilized pomace, in duplicate: three different biological samples.
4. Repassage over Aglianico fresh pomace: three different biological samples.
5. Enological activated carbon treatment: a total of 5 L of Primitivo stored in a steel tank containing 1.5 g of enological activated carbon, which was stirred for few seconds and left in static conditions for 24 h: three biological samples.

Thus, a total of 14 Primitivo red wine biological samples, as listed in Table 1, were obtained and stored in a cellar until analysis.

For the stabilized grape pomace, a total of 150 kg of Primitivo di Gioia grape pomaces were stored at room temperature for two months in 50 L steel tanks before being used for the repassage experiments. For the fresh grape pomace, a total of 250 kg of Primitivo and 250 kg of Aglianico pomaces were used fresh for the repassage experiments. The Primitivo di Gioia wine and grape pomace were purchased from a winery in Gioia del Colle (Apulia region, Italy). The Aglianico grape pomace was purchased in a winery in Nova Siri (Lucania region, Italy).

The determination of the total polyphenols (gallic acid content) and anthocyanins was carried out according to the International Organization of Vine and Wine (OIV) methods [34] and is reported in Supplementary Table S1. In particular, according to the OIV-MA-AS2-10 method, the spectrophotometric Folin–Ciocalteu assay was used to estimate the total polyphenol content (TPC) [35]. First, 1 mL of the wine, previously diluted 1/5, 50 mL of distilled water, 5 mL of Folin-Ciocalteu reagent, and 20 mL of sodium carbonate solution were introduced into a 100 mL flask. Distilled water was used to bring the volume up to 100 mL as a final volume. After mixing to dissolve all the contents, the solution was left for 30 min for the reaction to stabilize. The absorbance at 750 nm was determined through a path length of 1 cm compared with a blank prepared with distilled water in place of the wine. Gallic acid equivalents, or mg GAE, were used to express the results per liter of wine. For the anthocyanin content, the OIV-MA-AS315-11 method, involving the HPLC determination of nine major anthocyanins in red and rosé wine (type-II method) was used. The analysis of the wine was performed by direct separation by HPLC with the reverse phase column, gradient elution by water/formic acid/acetonitrile, and detection at 518 nm. The HPLC analysis was performed according to the following conditions: injection volume: 50 µL (red wine); flow: 0.8 mL/min; temperature: 40 °C; run time: 45 min; post run time: 5 min; detection: 518 nm. Malvidin chloride content (mg/L) values were used to express the results.

### 2.3. Repassage of Red Wines over Grape Pomace from Different Varieties

The prototype (Figure S1) and the process used in this study for the repassage experiments were those previously developed and optimized to remove OTA from contaminated wine by repassing the contaminated wine over uncontaminated grape pomaces [13,16]. The prototype [16] mainly consists of a 1000 L steel tank containing the wine to be repassed over the pomaces, 3 steel tanks, each filled with 73 kg of pomace, and a system of circulating pumps, sensors, and a control unit that makes the whole repassage process automated. The process used by the prototype was originally developed to maximize the removal of OTA during the repassage in the shortest possible time [16]. In particular, an aliquot of wine is pumped into the first tank until it covers the pomaces, left for 10 min, pumped in the second tank, left for 10 min, pumped in the third tank, left for 10 min, and then pumped into a collecting tank. As soon as the wine is transferred from the first tank to the second tank, a new amount of wine is added to the first tank and so on to continue the process. The process is completed when all the wine (~1000 L) has been repassed. The treatment with the enological activated carbon was performed in static conditions in a steel tank containing 5 L of Primitivo and 1.5 g of enological activated carbon, stirred for few seconds, and left in static conditions for 24 h.

### 2.4. NMR Measurements

The protocol applied for the wine samples’ preparation and NMR acquisition and analysis was according to the Bruker standard procedure established for the NMR screening of wine (Bruker BioSpin GmbH, Rheinstetten, Germany). The sample preparation and measurement protocol facilitates and optimizes the quantitative determination of metabolites and statistical analysis [24,36]. For each sample, 900 µL of wine was added to 100 µL of buffer (KH_2_PO_4_, NaN_3_ in D_2_O + 0.03% *v*/*v* 3-trimethylsilyl-propionic-2,2,3,3-d4 acid sodium salt, TSP). In order to minimize possible variations in the chemical shifts of signals among the samples, the pH values for the wines were adjusted (±0.02) to a wine reference value measured at 3.74, as reported in Table 1. This value is intermediate between the measured pH obtained for all the samples studied (ranging from 3.9 to 3.5) and is close to the reported value found for Primitivo wines [37]. Then, a total of 600 μL of the resulting solution was transferred into a 5 mm NMR tube for spectral acquisition. All the NMR spectra were recorded at a temperature of 300.0 K (±0.05), after 5 min for thermal equilibration, on a Bruker Avance III spectrometer (Bruker, Karlsruhe, Germany), operating at 400.13 MHz for ^1^H observation. Automated tuning and matching, locking, shimming, and calibration of the 90° hard pulse P (90°), including adjustment of the 25 Hz presaturation pulse, was performed for each sample using the standard Bruker routines (ATMA, LOCK, TOPSHIM, and PULSECAL, respectively) to optimize the NMR conditions [36]. Two ^1^H NMR experiments were performed for each sample in the automation procedure (zgpr.mod and noesygpps1d.comp1 Bruker pulse sequences). The measurements were repeated once in random order after the completion of the first entire set. The automated noesygpps1d.comp1 pulse sequence was used to enhance the minor compound signals [26,38,39,40]. The AQ parameters were as follows: 32 scans, 64K data points, a spectral width of 8223.685 Hz, an acquisition time of 3.98 s, a relaxation delay of 4 s, and a mixing time of 10 ms. The FIDs were multiplied by an exponential weighting function corresponding to a line broadening of 0.3 Hz before Fourier transformation, phasing, and base line correction. The metabolites were assigned on the basis of analysis of 2D NMR spectra (2D ^1^H Jres, ^1^H COSY, ^1^H-^13^C HSQC, and HMBC, which were also acquired for assignment purposes) and compared with the published data [22,29,36,38,39,40].

### 2.5. Multivariate Data Analysis

The NMR spectra were processed using Topspin 3.6.5 and visually inspected using Amix 3.9.13 (Bruker, Biospin, Italy). The ^1^H NMR (noesygpps1d.comp1 Bruker pulse sequence) spectra were segmented into rectangular buckets of a fixed 0.04 ppm width and integrated. In order to reduce the number of variables (assigned/not assigned NMR signals) and to compensate for small shifts in the positions of the peaks, the NMR spectra were converted into data matrices through a bucketing procedure. By this method, the integration of the NMR signals into small spectral regions, called “buckets” or “bins”, was performed. Simple equidistant binning is the most commonly used method, and it has already been described as a robust procedure in metabolomic fingerprinting for sample classification [41,42]. The spectral regions between 4.90 and 4.75, 3.70 and 3.60, and 1.22 and 1.15 ppm were discarded because of the residual peaks of water and ethanol signals (in particular, the quartet at 3.66 ppm, the -CH_2_ group and the triplet at 1.18 ppm, and the-CH_3_ group of ethanol). The resulting data sets consisted of the 228 variables (^1^H NMR spectra bucketed values, in columns) measured for each wine sample (row). The description of the statistical analyses refers to Pareto scaled data (performed by dividing the mean-centered data by the square root of the standard deviation) [43]. The data table generated from all 16 spectra was considered for multivariate data analysis (MVA) by using Simca-P version 14 (Sartorius Stedim Biotech, Umea, Sweden). In particular, unsupervised (principal component analysis (PCA)) and supervised (partial least squares discriminant analysis (PLS-DA) and orthogonal partial least squares discriminant analysis (OPLS-DA)) methods were performed to analyze the intrinsic variation in the data [44,45,46]. The models validation was performed by using the internal cross-validation default method (7-fold) and permutation test (400 permutations). The quality of the models was evaluated by the R^2^ and Q^2^ parameters. The first (R^2^) is a cross-validation parameter defined as the data variance portion explained by the model and specifies goodness-of-fit. The second (Q^2^) describes the portion of data variance predictable by the model [44,46,47,48]. The S-line plot for the OPLS-DA models visualizes the centered loading vector p (ctr), colored according to the correlation loading absolute value, p(corr). The loading line plot for the PLS-DA model displays the correlation loading as the structure between X and Y (w*c) [45].

## 3. Results

### 3.1. ^1^H NMR Spectroscopy

**Figure 1:**
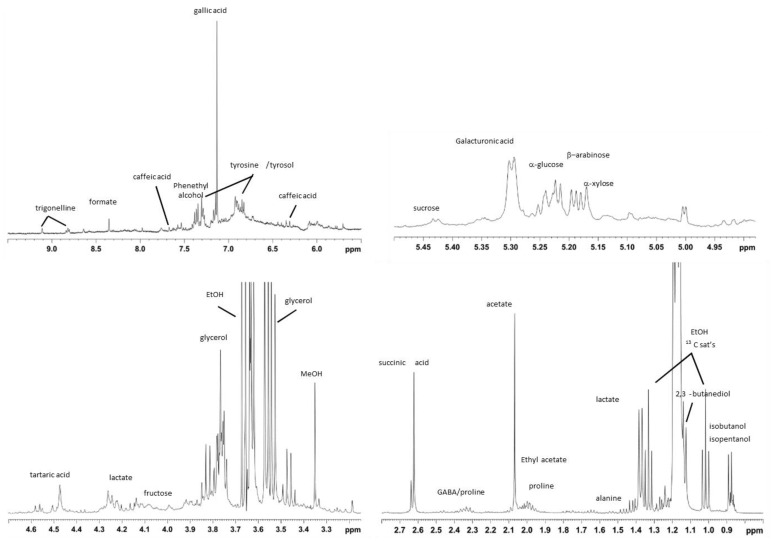
^1^H NMR relative expansions of significant spectral regions (400 MHz, D_2_O) of a specimen of analyzed wine spectra, acquired with automated noesygpps1d.comp1 Bruker sequence.

The relative expansions of the regions of the representative studied Primitivo wine ^1^H-NMR spectrum and the peak assignments of the relevant metabolites carried out by a comparison of literature data [22,36,38,39,40] and bi-dimensional experiments are reported in Figure 1. Besides the highest peaks, assigned to the -CH_3_ ethanol signal, the presence of other alcohols (isobutanol, isopentanol, 2,3 butanediol) as well as aliphatic groups of amino acid (alanine, proline, GABA) and organic acid (lactic, acetic, succinic) resonances were observed in the low-frequency field region of the spectrum (0.5–3.00 ppm). Intense peaks assigned to glycerol and resonances ascribable to tartrate, sucrose, and methanol were observed in the middle frequency region of the spectrum (3–5 ppm). Protons of sugars such as glucose, sucrose, xylose, arabinose, and galacturonic acid were also observed in the 5–5.5 ppm spectral region. Finally, in the aromatic spectral region (5.5–9.5 ppm), proton resonances of caffeic acid, tyrosol, gallic acid, tyrosine, phenethyl alcohol, formic acid, and trigonelline were identified. A summary of the identified metabolites is reported in Table S2. The whole set of 14 NMR spectra for all the studied samples is reported in Figure S2.

### 3.2. Multivariate Analysis

**Figure 2:**
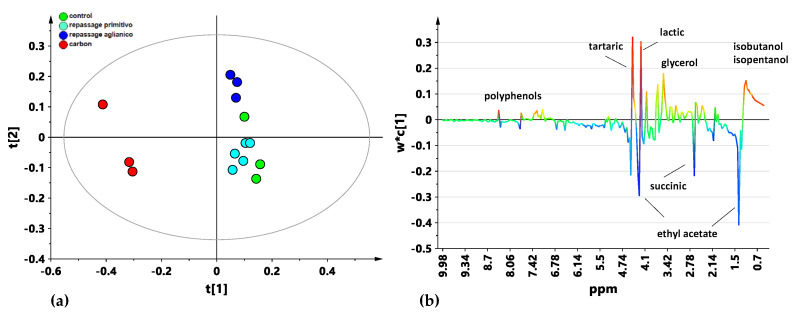
(**a**) PLS-DA t [1]/t [2] score plot (two components, R^2^X = 0.59, R^2^Y = 0.53, Q^2^ = 0.20) for the whole wine sample data set, classified according to the performed OTA decontamination treatment: Primitivo grape pomace repassage (sky-blue circle), Aglianico grape pomace repassage (blue circle), activated carbon (red circle) and control samples (green circles). (**b**). Loading S-line plot for the PLS-DA model, colored according to the correlation scaled coefficient. The *x*-axis indicates binned signals (ppm) in the ^1^H NMR spectrum.

The first level of investigation was carried out using the supervised PLS-DA (Figure 2) methods, on the whole bucket-reduced spectra in the 0.5–10 ppm spectral range [49]. The application of these methods to the spectral dataset allowed us to obtain information on both the general trends of the data and the discriminating metabolites responsible for the class grouping [50]. The samples obtained from the repassage over the stabilized and fresh Primitivo pomace were further considered as a single class. As shown in the t [1]/t [2] PLS-DA score plot (two components, R^2^X = 0.59, R^2^Y = 0.53, Q^2^ = 0.20) in Figure 2a, a certain degree of separation was found among the sample classes. In particular, the wines treated with the activated carbon were clearly separated along the first component t [1] from all the other repassed wine classes (repassage over fresh/stabilized Primitivo pomace and repassage over fresh Aglianico pomace) and the control samples. This could suggest that the metabolic profiles of the activated-carbon-treated samples exhibited qualitative chemical composition different from the grape-repassed and untreated samples. Moreover, it should be noted that the control wine samples (Primitivo wine not repassed over pomace) results show that it is clearly separated on the second component t [2] from the grape-repassed ones. The metabolites responsible for the observed separation of the activated-carbon-treated samples compared with the other wine classes (both controls and repassed over Primitivo and/or Aglianico pomace) were seen in the S-line plot for the model, as reported in Figure 2b. Relative higher levels of specific metabolites such as ethyl acetate and succinate were seen in the activated-carbon-treated samples, while relatively higher levels of glycerol, lactate, tartaric, isobutanol, isopentanol, and polyphenol content were found in the remaining samples.

**Figure 3:**
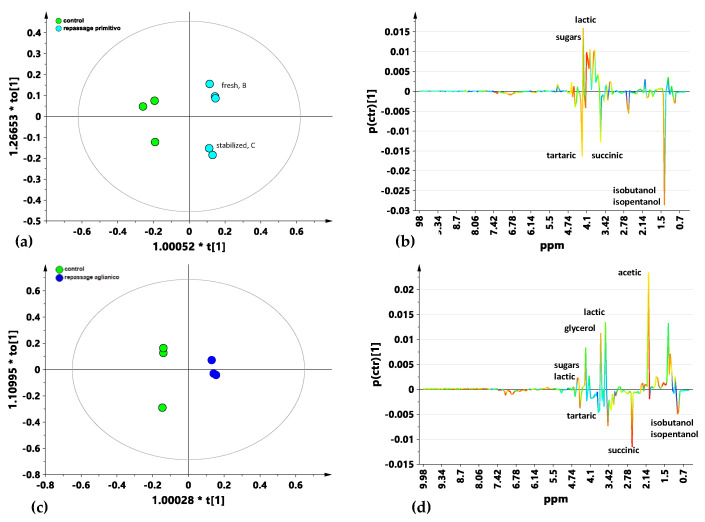
Pairwise OPLS-DA t [1]/t [2] score plots for untreated (controls) vs. (**a**) Primitivo (1 + 2 + 0; R^2^X = 0.82, R^2^Y = 0.98 and Q^2^ = 0.83) and (**c**) Aglianico (1 + 2 + 0; R^2^X = 0.84, R^2^Y = 0.99 and Q^2^ = 0.91) repassed wine samples. Sample symbols are colored according to the different OTA decontamination treatment: control, green circle; Aglianico, blue circles; and Primitivo, green circles. Metabolites responsible for the class separation can be observed in the model-related S-line plots, (**b**,**d**) colored according to the correlation scaled coefficient. The *x*-axis indicates binned signals (ppm) in the ^1^H NMR spectrum.

In order to further analyze the wine metabolic response due to the repassage over the pomace using different grape varieties (Primitivo and Aglianico), the metabolic profiles of the treated and untreated classes were studied by supervised OPLS-DA pairwise comparisons. Two different OPLS-DA models were built, comparing separately, against the control group, the wines with repassage over fresh/stabilized Primitivo (Figure 3a,b) and over fresh Aglianico pomace (Figure 3c,d), respectively. This was carried out in order to ascertain whether the grape variety could have a different effect on the processed wine samples’ characteristics. The OPLS-DA models were both obtained by using one predictive and two orthogonal components (1 + 2 + 0). Good model quality parameters were also found with R^2^X = 0.82, R^2^Y = 0.98 and Q^2^ = 0.83 for the controls vs. samples treated with Primitivo pomace repassage (Figure 3a) and R^2^X = 0.84, R^2^Y = 0.99 and Q^2^ = 0.91 for the controls vs. samples treated with Aglianico pomace repassage (Figure 3c) OPLS-DA, respectively. As shown by the limited dispersion along the first orthogonal component (Figure 3a), the samples obtained from repassage over the Primitivo stabilized pomace did not differ significantly from the samples with repassage over the fresh Primitivo pomace, confirming the original PLS-DA results. This aspect could be of interest for wineries, since stabilized pomace can be recovered over time and reused for repassage with efficacy [13]. A clear difference was also observed in the OPLS-DA model shown in Figure 3c, comparing the samples obtained by repassage over the Aglianico pomace with the control group. From the corresponding S-line plots for both the OPLS-DA models, the molecular components, distinctive for each class and responsible for differentiation with the controls, were identified (Figure 3b,d). Interestingly, the discriminating metabolites between the controls vs. Primitivo and Aglianico grape-pomace-repassed samples appear to be similar (Figure 3b,d). Thus, in both pairwise comparison, the untreated control wines were shown to be characterized by a higher relative content of organic acids, such as tartaric and succinic acids, and containing isopentanol as well as isobutanol. On the other hand, the Aglianico and Primitivo grape-pomace-treated samples showed a higher relative content of sugars and lactic acid. Moreover, differently from the wines repassed over the Primitivo pomace, the Aglianico repassed samples were also characterized by a higher relative amount of glycerol and acetic acid. This could suggest that the usage of different pomace grape varieties may lead to a certain degree of specific quality modification in the final product.

**Figure 4:**
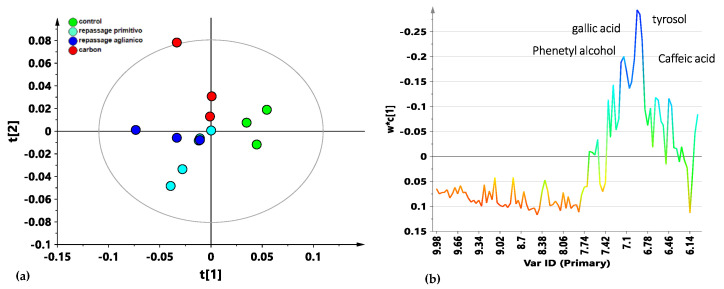
(**a**) Focused aromatic spectral region PLS-DA t [1]/t [2] score plot (five components, R^2^X = 0.92, R^2^Y = 0.85, Q^2^ = 0.56) for the wine samples, classified according to the performed OTA decontamination treatment: Primitivo grape pomace repassage (sky-blue circle), Aglianico grape pomace repassage (blue circle), activated carbon (red circle) and control samples (green circles). (**b**) Metabolites responsible for the class separation could be observed in the model-related loading line plots, colored according to the correlation scaled coefficient. The *x*-axis indicates binned signals (ppm) in the ^1^H NMR spectrum. Quality parameters (Correct Classification Rate), CCR; area under the Receiver Operating Characteristic (ROC) curve (AUC); intercepts of R^2^ and Q^2^ values on the *y*-axis; Fisher’s probability index) of the model are reported in Table S3.

Finally, a selected bucketing from the ^1^H 1D-NOESY spectra was further considered, focusing only on the aromatic spectral region (6.00–10.00 ppm). From the PLS-DA t [1]/t [2] score plot visual inspection (five components, R^2^X = 0.92, R^2^Y = 0.85, Q^2^ = 0.56, Figure 4a), the control samples were well separated from all the other samples along the first component t [1]. The activated-carbon-treated samples were shown to be clearly separated on the second component t [2], both from the controls and from all the wines obtained by repassage over the different varieties of grape pomace. The loading line plot of the model (Figure 4b) revealed the binned signals assigned to the functional groups of the aromatic molecules responsible for class discrimination. The phenol content was shown to be generally higher in the controls than in the other treated classes. Specifically, a higher content of gallic and caffeic acids, phenethyl alcohol, and tyrosol could be seen for the control samples compared with the grape-repassed and carbon-treated samples.

**Figure 5:**
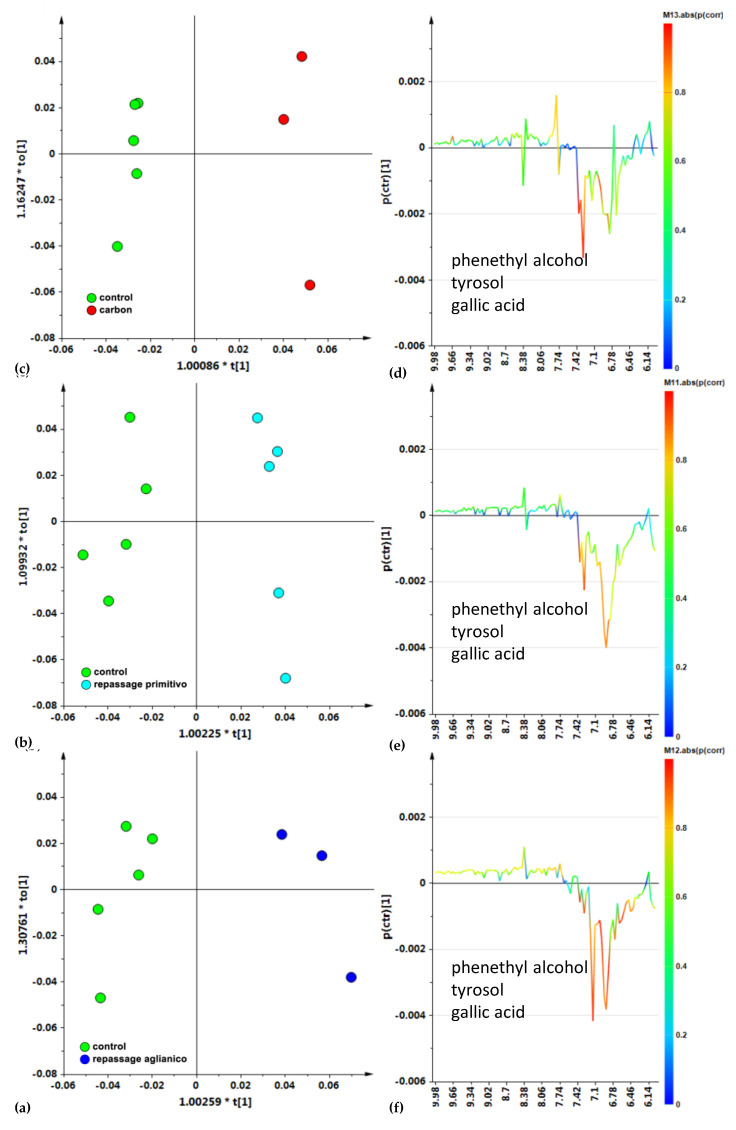
Focused aromatic spectral region OPLS-DA t [1]/to [1] score plot, for the control vs. (**a**) activated carbon (1 + 1 + 0; R^2^X = 0.69, R^2^Y = 0.99, Q^2^ = 0.96); (**b**) Primitivo pomace (1 + 1 + 0; R^2^X = 0.79, R^2^Y = 0.96, Q^2^ = 0.90) and (**c**) Aglianico pomace (1 + 1 + 0; R^2^X = 0.78, R^2^Y = 0.94, Q^2^ = 0.83) wine samples. Sample symbols are colored according to the different OTA decontamination treatment: control, green circle; Aglianico, blue circles; and Primitivo green circles. Metabolites responsible for the class separation can be observed in the OPLS-DA model-related loading S-line plots (**d**–**f**), colored according to the correlation scaled coefficient. The *x*-axis indicates binned signals (ppm) in the ^1^H NMR spectrum.

A supervised pairwise OPLS-DA analysis was then performed, obtained by focusing on the aromatic spectral region (Figure 5), to further investigate the metabolites responsible for the partition of the different classes. A clear partition between the controls vs. Aglianico (Figure 5a) grape-repassed, Primitivo (Figure 5b) grape-repassed, and activated-carbon-treated (Figure 5c) samples can be observed in the related score plot. Moreover, the S-line plot for the models showed that a higher relative content of phenolic compounds characterized the control wine samples, in comparison with both the carbon-treated (Figure 5d), Primitivo and Aglianico (Figure 5e) grape-pomace-repassed wines (Figure 5e) and Aglianico (Figure 5f) pomace samples. In particular, the molecules responsible for discrimination among the samples were shown to be tyrosol (bins at 6.94 and 7.18 ppm), caffeic acid (bin at 6.46 ppm), and phenethyl alcohol (bins at 7.30 and 7.38 ppm) together with gallic acid (bin at 7.14 ppm). A lower relative content of gallic acid was found in particular for the samples with repassage over the Aglianico pomace compared with the controls (Figure 5f). These results for the metabolite contents are consistent with previously obtained classical data and are reported in Supplementary as Table S1.

## 4. Discussion

Multivariate statistical analyses were applied to the NMR-based metabolomics data of the Primitivo wine samples. These resulted from OTA decontamination treatments based on repassage over Primitivo and/or Aglianico pomace and activated carbon treatments compared with untreated Primitivo wines (controls). The analyses allowed the characterization of the metabolic response to the decontamination treatment in the processed products. Relatively higher levels of specific metabolites, such as ethyl acetate and succinate, were found in the activated-carbon-treated samples compared with all the otherwise-processed samples, including the controls. Ethyl acetate, one of the main contributors to volatile acidity, is the most abundant ester in wine and it also constitutes an oxidation indicator, since it is responsible for acescency, a typical alteration of sensory properties [51]. However, at concentrations below 80 mg/L, it could also positively influence the wine’s aroma [52,53,54]. Succinic acid is one of the main organic acids, formed during alcoholic fermentation, and its level varies between grape cultivars, with higher concentrations in red grape [55]. Succinic acid is one of the most important non-volatile acids in wine, and it was reported to be responsible for the largest part of the increase in titratable acidity [56,57]. Although its production can be affected by alcohol concentration, the observed succinic acid content in wines is very stable, since it does not change over aging. The organoleptic character of succinic acid has been described as sour with a salty, bitter taste. Due to its bitter, salty flavor, winemakers watch out for the levels of succinic acid in wine [57]. On the contrary, in all the grape-pomace-repassed wine samples, relatively higher contents of glycerol, lactate, tartaric, isobutanol, isopentanol, and phenolic compounds were found compared with the activated-carbon-treated samples. Like succinic acid, tartaric acid is one of the major contributors to wine acidity and it influences the perception of a tart taste in wine [58]. The level of tartaric acid is strictly related to the cultivar, ripening stage, and maturity, and it may be used as a biomarker for characterizing grape cultivars. Nevertheless, it should be also considered that its content can be significantly altered depending on the levels of potassium bitartrate and calcium tartrate formation, which precipitate in wines [59]. In addition, the precipitation process is also dependent on the winemaking conditions, including temperature, pH, and the concentration of calcium or potassium [60]. Also, lactic acid contributes to the wine total acidity and, unlike malic and tartaric acid, being a softer and milder acid, its level is associated with a creamier mouthfeel of wine [57]. Glycerol is one of the most abundant components of wine and its content has been associated with several attributes of taste, such as oiliness, persistence, and mellowness in the mouth [61]. Thus, glycerol plays a significant role in the organoleptic properties of wine, although an overproduction of this compound is usually linked with an acetic acid accumulation [60]. Finally, a relatively high content of isobutanol and isopentanol was selectively found in the control wine samples compared with the repassed wine samples. These molecules (isobutanol and isopentanol) were generally identified as wine higher alcohols and contribute to assessing the aromatic sensory perception of red wine aroma (fruity, flowery notes and aroma complexity) [62,63]. Finally, the multivariate analysis focused on the aromatic spectral region showed that, consistent with the literature data, carbon treatment induces a marked decrease in the polyphenol content of wine [64], thus affecting the flavor, color, and bouquet of the final product [65,66]. In fact, as described in the literature, the removal of OTA through the use of fining agents such as carbon is based on a non-specific adsorption mechanism, which causes a decrease in the content of phenols, flavonoids, total anthocyanins, and polymeric pigments [64]. Moreover, the presence of relatively higher levels of glycerol, lactate, tartaric, isobutanol, isopentanol, and phenols, observed in all the otherwise-processed samples, compared with the activated carbon treatment samples, strongly supports the possible use of pomace for OTA decontamination without significantly affecting the organoleptic properties of wine. Indeed, the ^1^H NMR profile-based data analyses suggest that treatment with activated carbon is definitely more invasive compared with those samples treated with pomace for OTA removal. Although some decrease in specific metabolites was also observed compared with the controls for the pomace-based processes, the activated carbon treatment produced a general impoverishment of the metabolomic profiles of the studied wines. Moreover, as already reported in the literature, despite high efficiency, the OTA detoxification of wine, by the use of activated carbon fining adsorbents, causes a modification of organoleptic properties even when used at the recommended dosage range [4,64]. The alternative technique of repassage over pomace could be therefore be a successful method for the removal of OTA. Interestingly, although a certain degree of differentiation in the final product was observed, the wine samples obtained by repassage over the pomace from different grape varieties did not show remarkable differences. At the same time, it should be also highlighted that the usage of stabilized compared with fresh pomace did not show major differences, suggesting the possible use of stored material as a useful tool for modern wineries.

## 5. Conclusions

In this work, a ^1^H-NMR-based metabolomic method was applied to a wine data set with the aim of identifying any possible variation in the chemical profiles after different OTA removal treatments. In particular, the untreated (control) Primitivo wine sample metabolic profiles were compared with Primitivo fresh/stabilized and Aglianico grape-pomace-repassed and activated-carbon-treated wines. As shown in the results of the statistical analysis, the metabolic profiles of the activated-carbon-treated wine samples exhibited a decrease in important quality-related metabolites such as glycerol, lactic, and tartaric acids, isobutanol/pentanol, and polyphenols compared with the untreated and grape-pomace-repassed wine samples. Moreover, although a lower content of phenolic compounds was observed in the pomace-based processed wines compared with the control samples, our findings suggest the repassage over pomace could be a successful method for efficient OTA removal without significantly altering the chemical profile and related oenological characteristics. Thus, the alternative technique of repassage over pomace may be proposed as a natural adsorbing/decontaminating material for the removal of OTA.

## Figures and Tables

**Table 1:** List of analyzed control/OTA-removal-treated wine samples and related initial and reference adjusted pH values.

NMR ID	Treatment	Wine	Initial pH	Final pH * t
A	Control (absence of OTA)	Primitivo di Gioia	3.74	3.74
A	Control (absence of OTA)	Primitivo di Gioia	3.66	3.74
A	Control (absence of OTA)	Primitivo di Gioia	3.62	3.72
B	Repassage over fresh Primitivo pomace	Primitivo di Gioia	3.69	3.72
B	Repassage over fresh Primitivo pomace	Primitivo di Gioia	3.68	3.74
B	Repassage over fresh Primitivo pomace	Primitivo di Gioia	3.68	3.73
C	Repassage over stabilized Primitivo pomace	Primitivo di Gioia	3.67	3.73
C	Repassage over stabilized Primitivo pomace	Primitivo di Gioia	3.69	3.75
D	Repassage over fresh Aglianico pomace	Blend Primitivo di Gioia and Primitivo di Manduria	3.55	3.75
D	Repassage over fresh Aglianico pomace	Blend Primitivo di Gioia and Primitivo di Manduria	3.59	3.74
D	Repassage over fresh Aglianico pomace	Blend Primitivo di Gioia and Primitivo di Manduria	3.61	3.74
E	Activated carbon	Primitivo di Gioia	3.83	3.74
E	Activated carbon	Primitivo di Gioia	3.70	3.74
E	Activated carbon	Primitivo di Gioia	3.90	3.74

* After adjustment to pH wine reference (±0.02).

## Data Availability

The original contributions presented in this study are included in the article/Supplementary Materials. Further inquiries can be directed to the corresponding author.

## Supplementary Materials

The following supporting information can be downloaded at: https://www.mdpi.com/article/10.3390/foods14050734/s1, Figure S1. Prototype scheme (a,b) photo of must/wine decontamination. Figure S2. ^1^H NMR spectrum (noesygppsld.compl pulse program) of (a–c) control; (d–f) Primitivo fresh grape pomace; (g,h) Primitivo stabilized grape pomace; (i–k) Aglianico fresh grape pomace; (l–n) Activated-carbon-treated wine samples. Table S1. Mean and standard deviation of total polyphenols (gallic acid content) and anthocyanins (malvin chloride) of wine samples (mg/L). Table S2. ^1^H-NMR signals (chemical shifts) of assigned metabolites in wine spectra. Table S3. Performance and validation results to assess accuracy and robustness of PLS-DA model (Figure 4) for discrimination of untreated and treated wine samples, focusing on aromatic spectral regions.
