# Supplementary material for: 1H NMR Spectroscopy Primitivo Red Wine Screening After Grape Pomace Repassage for Possible Toxin Contamination Removal
Source: Foods. 2025 Feb 21;14(5):734. doi: 10.3390/foods14050734 (PMC11898919; doi:10.3390/foods14050734)
Supplement: Supplementary file 1 [file foods-14-00734-s001.zip › foods-3446744-supplementary.pdf]

foods

MDPI

Supplementary material

^{1}H NMR Spectroscopy Primitivo red wine screening after grape pomace repassage for possible toxins contamination removal

Laura Del Coco [1], Chiara Roberta Girelli[1], Lucia Gambacorta [2], Michele Solfrizzo [2], Francesco Paolo Fanizzi [1,*]

[1] Department of Biological and Environmental Science and Technology, University of Salento, via Lecce- Monteroni, 73100, Lecce, Italy; chiara.girelli@unisalento.it (C.R.G.)

[2] Institute of Sciences of Food Production (ISPA), National Research Council (CNR), Via Amendola 122/O, 70126 Bari, Italy; lucia.gambacorta@ispa.cnr.it (L.G.), michele.solfrizzo@ispa.cnr.it (M.S.)

* Correspondence: fp.fanizzi@unisalento.it

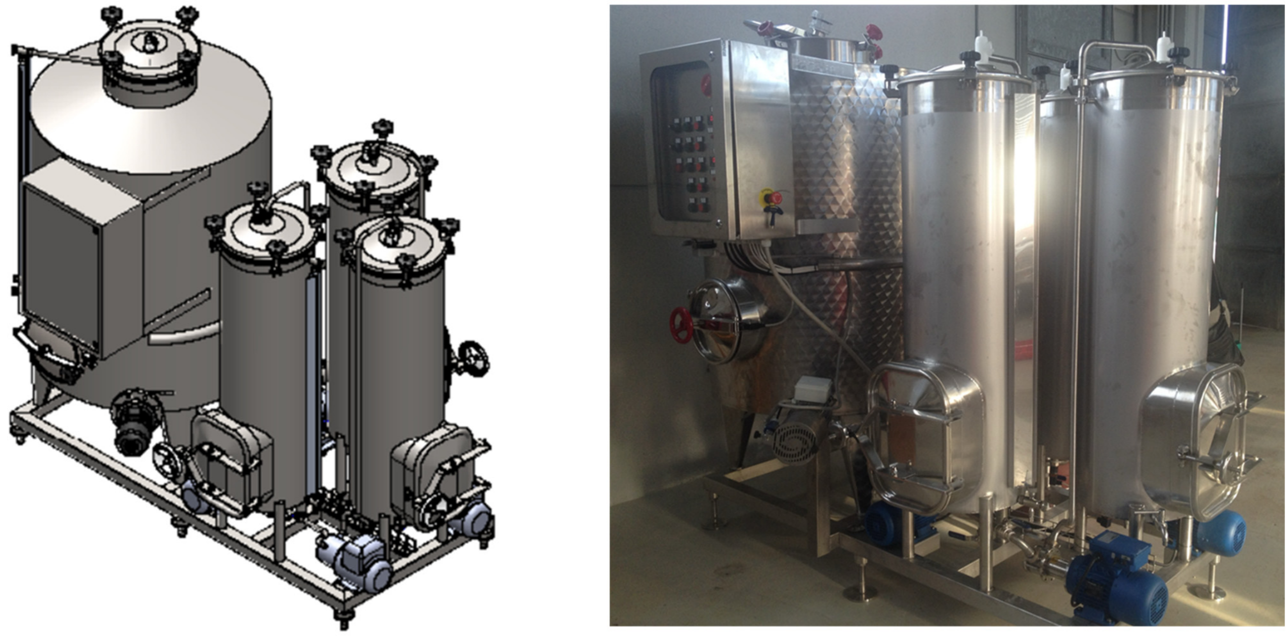

a) b)

Figure S1: Prototype scheme **a)** and photo **b)** of Must/Wine Decontamination.

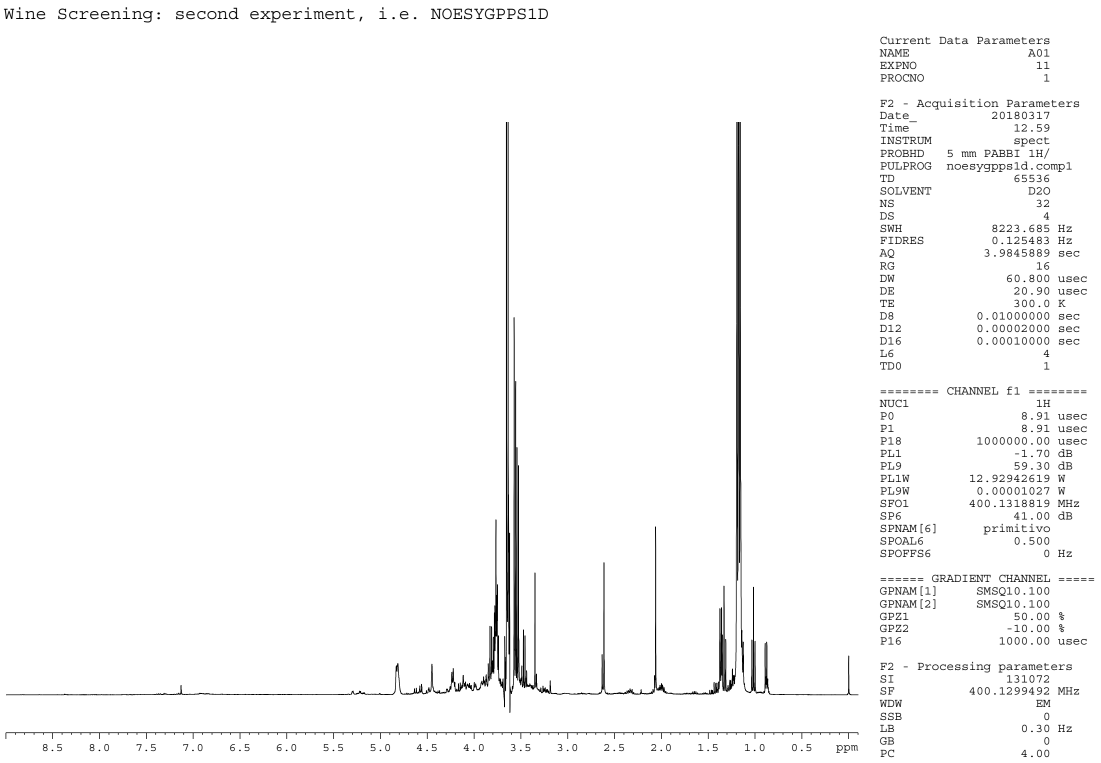
Wine Screening: second experiment, i.e. NOESYGPPS1D
8.5 8.0 7.5 7.0 6.5 6.0 5.5 5.0 4.5 4.0 3.5 3.0 2.5 2.0 1.5 1.0 0.5 ppm
Current Data Parameters
NAME A01
EXPNO 11
PROCNO 1
F2 - Acquisition Parameters
Date_ 20180317
Time 12.59
INSTRUM spect
PROBHD 5 mm PABBI 1H/
PULPROG noesygppps1d.comp1
TD 65536
SOLVENT D2O
NS 32
DS 4
SWH 8223.685 Hz
FIDRES 0.125483 Hz
AQ 3.9845889 sec
RG 16
DW 60.800 usec
DE 20.90 usec
TE 300.0 K
D8 0.01000000 sec
D12 0.00002000 sec
D16 0.00010000 sec
L6 4
TD0 1
======== CHANNEL f1 ========
NUC1 1H
P0 8.91 usec
P1 8.91 usec
P18 1000000.00 usec
PL1 -1.70 dB
PL9 59.30 dB
PL1W 12.92942619 W
PL9W 0.00001027 W
SFO1 400.1318819 MHz
SP6 41.00 dB
SPNAM[6] primitivo
SPOAL6 0.500
SPOFFS6 0 Hz
====== GRADIENT CHANNEL =====
GPNAM[1] SMSQ10.100
GPNAM[2] SMSQ10.100
GPZ1 50.00 %
GPZ2 -10.00 %
P16 1000.00 usec
F2 - Processing parameters
SI 131072
SF 400.1299492 MHz
WDW EM
SSB 0
LB 0.30 Hz
GB 0
PC 4.00

a)

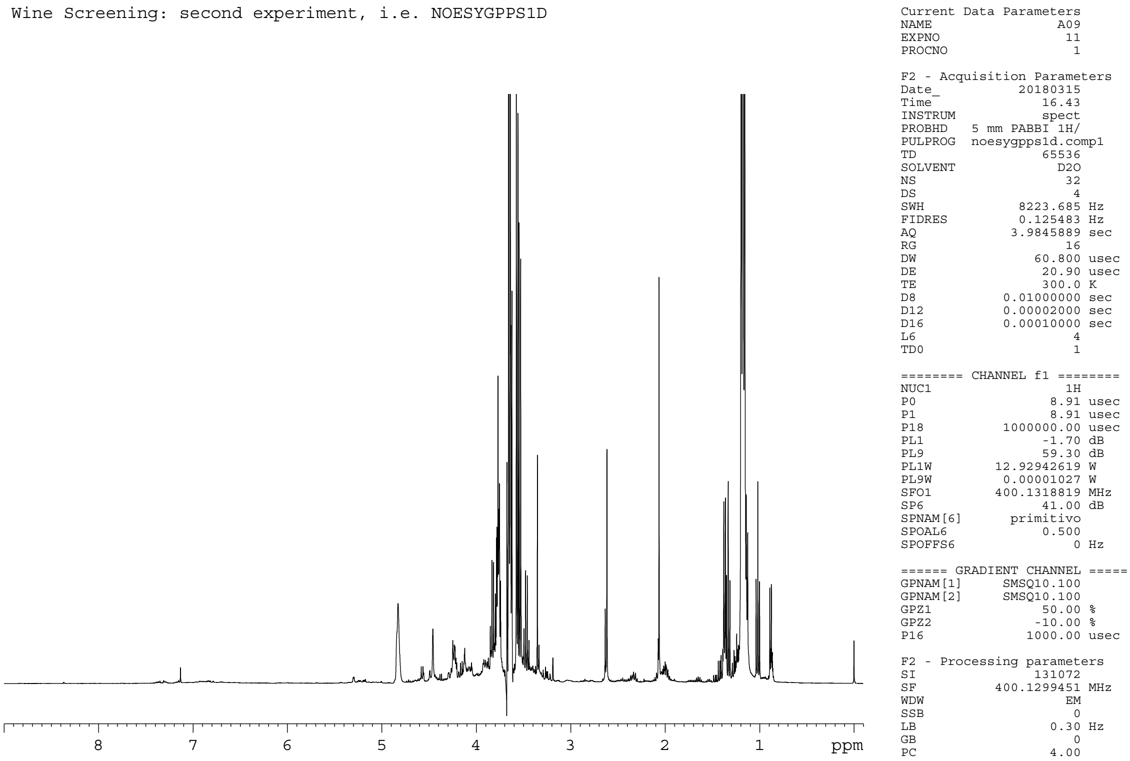
Wine Screening: second experiment, i.e. NOESYGPPS1D
8 7 6 5 4 3 2 1 ppm
Current Data Parameters
NAME A09
EXPNO 11
PROCNO 1
F2 - Acquisition Parameters
Date_ 20180315
Time 16.43
INSTRUM spect
PROBHD 5 mm PABBI 1H/
PULPROG noesygppps1d.comp1
TD 65536
SOLVENT D2O
NS 32
DS 4
SWH 8223.685 Hz
FIDRES 0.125483 Hz
AQ 3.9845889 sec
RG 16
DW 60.800 usec
DE 20.90 usec
TE 300.0 K
D8 0.01000000 sec
D12 0.00002000 sec
D16 0.00010000 sec
L6 4
TD0 1
======== CHANNEL f1 ========
NUC1 1H
P0 8.91 usec
P1 8.91 usec
P18 1000000.00 usec
PL1 -1.70 dB
PL9 59.30 dB
PL1W 12.92942619 W
PL9W 0.00001027 W
SFO1 400.1318819 MHz
SP6 41.00 dB
SPNAM[6] primitivo
SPOAL6 0.500
SPOFFS6 0 Hz
====== GRADIENT CHANNEL =====
GPNAM[1] SMSQ10.100
GPNAM[2] SMSQ10.100
GPZ1 50.00 %
GPZ2 -10.00 %
P16 1000.00 usec
F2 - Processing parameters
SI 131072
SF 400.1299451 MHz
WDW EM
SSB 0
LB 0.30 Hz
GB 0
PC 4.00

b)

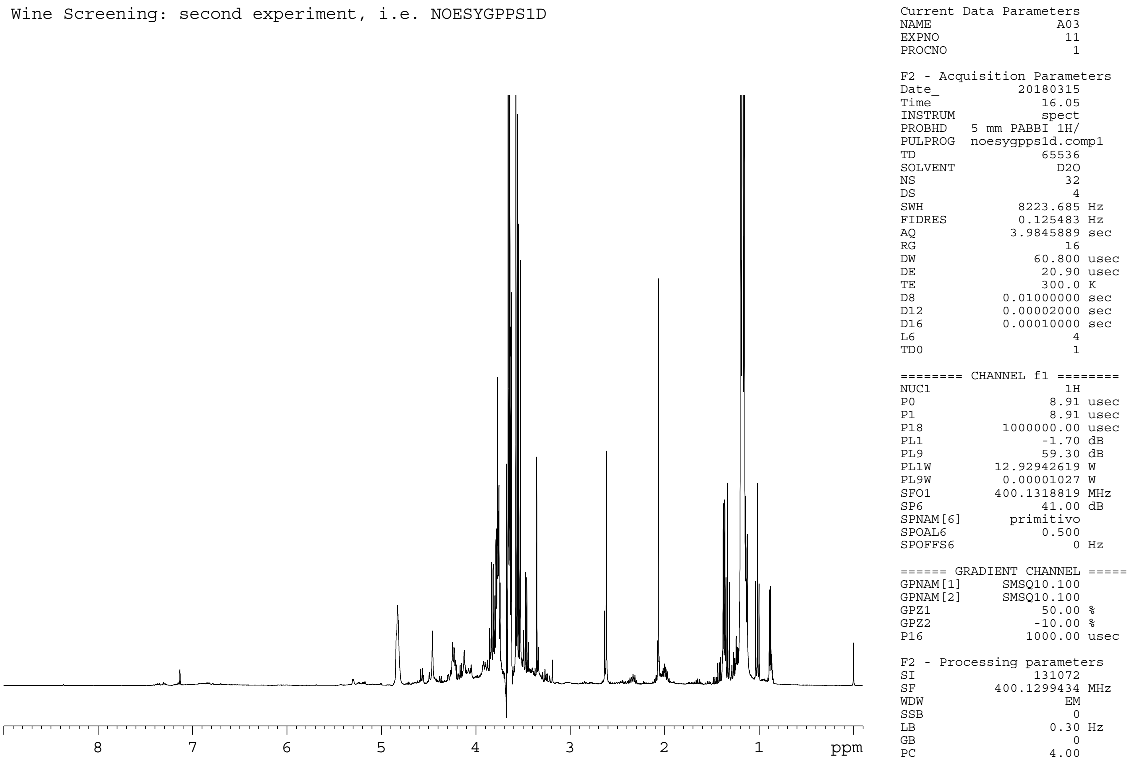
Wine Screening: second experiment, i.e. NOESYGPPS1D
8
7
6
5
4
3
2
1
ppm
Current Data Parameters
NAME A03
EXPNO 11
PROCNO 1
F2 - Acquisition Parameters
Date_ 20180315
Time 16.05
INSTRUM spect
PROBHD 5 mm PABBI 1H/
PULPROG noesygppps1d.comp1
TD 65536
SOLVENT D2O
NS 32
DS 4
SWH 8223.685 Hz
FIDRES 0.125483 Hz
AQ 3.9845889 sec
RG 16
DW 60.800 usec
DE 20.90 usec
TE 300.0 K
D8 0.01000000 sec
D12 0.00002000 sec
D16 0.00010000 sec
L6 4
TD0 1
======== CHANNEL f1 ========
NUC1 1H
P0 8.91 usec
P1 8.91 usec
P18 1000000.00 usec
PL1 -1.70 dB
PL9 59.30 dB
PL1W 12.92942619 W
PL9W 0.00001027 W
SFO1 400.1318819 MHz
SP6 41.00 dB
SPNAM[6] primitivo
SPOAL6 0.500
SPOFFS6 0 Hz
====== GRADIENT CHANNEL =====
GPNAM[1] SMSQ10.100
GPNAM[2] SMSQ10.100
GPZ1 50.00 %
GPZ2 -10.00 %
P16 1000.00 usec
F2 - Processing parameters
SI 131072
SF 400.1299434 MHz
WDW EM
SSB 0
LB 0.30 Hz
GB 0
PC 4.00

c)

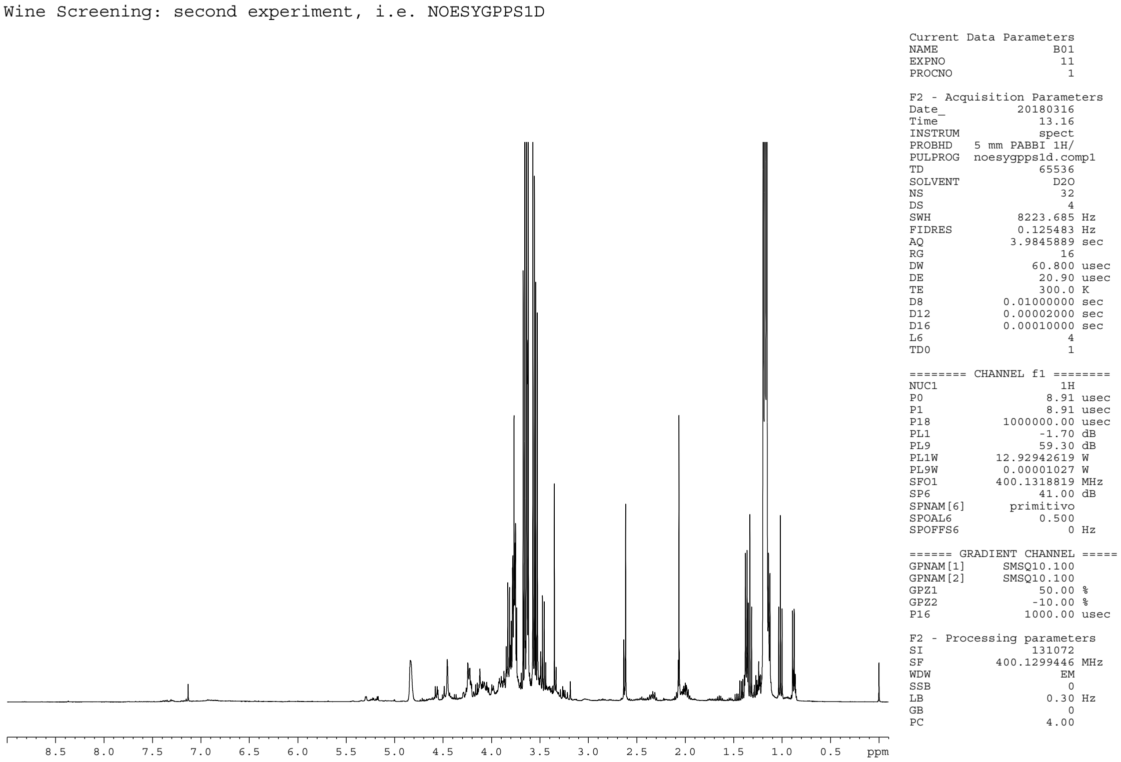
Wine Screening: second experiment, i.e. NOESYGPPS1D
8.5
8.0
7.5
7.0
6.5
6.0
5.5
5.0
4.5
4.0
3.5
3.0
2.5
2.0
1.5
1.0
0.5
ppm
Current Data Parameters
NAME B01
EXPNO 11
PROCNO 1
F2 - Acquisition Parameters
Date_ 20180316
Time 13.16
INSTRUM spect
PROBHD 5 mm PABBI 1H/
PULPROG noesygppps1d.comp1
TD 65536
SOLVENT D2O
NS 32
DS 4
SWH 8223.685 Hz
FIDRES 0.125483 Hz
AQ 3.9845889 sec
RG 16
DW 60.800 usec
DE 20.90 usec
TE 300.0 K
D8 0.01000000 sec
D12 0.00002000 sec
D16 0.00010000 sec
L6 4
TD0 1
======== CHANNEL f1 ========
NUC1 1H
P0 8.91 usec
P1 8.91 usec
P18 1000000.00 usec
PL1 -1.70 dB
PL9 59.30 dB
PL1W 12.92942619 W
PL9W 0.00001027 W
SFO1 400.1318819 MHz
SP6 41.00 dB
SPNAM[6] primitivo
SPOAL6 0.500
SPOFFS6 0 Hz
====== GRADIENT CHANNEL =====
GPNAM[1] SMSQ10.100
GPNAM[2] SMSQ10.100
GPZ1 50.00 %
GPZ2 -10.00 %
P16 1000.00 usec
F2 - Processing parameters
SI 131072
SF 400.1299446 MHz
WDW EM
SSB 0
LB 0.30 Hz
GB 0
PC 4.00

d)

Wine Screening: second experiment, i.e. NOESYGPPS1D

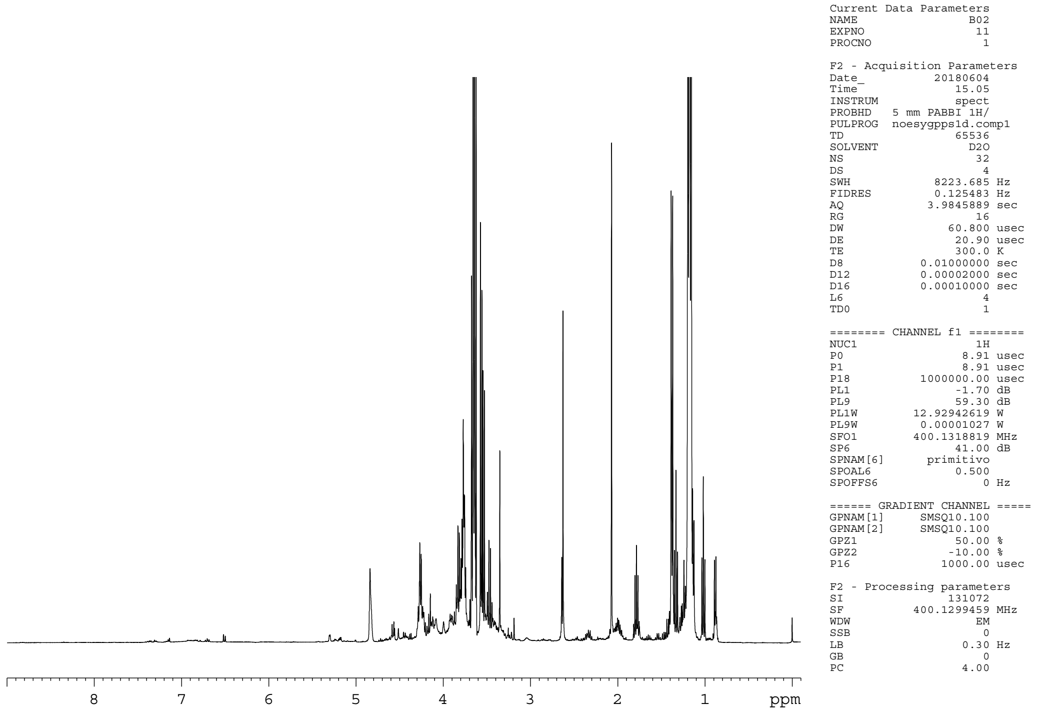

e)

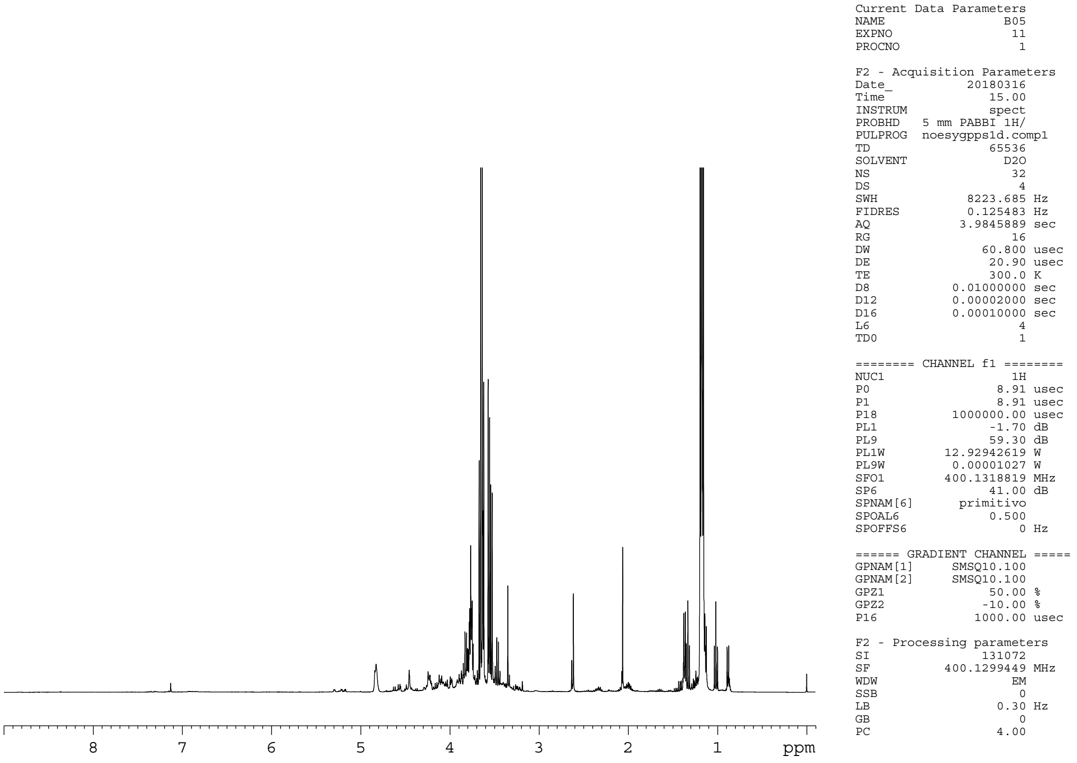

f)

Wine Screening: second experiment, i.e. NOESYGPPS1D

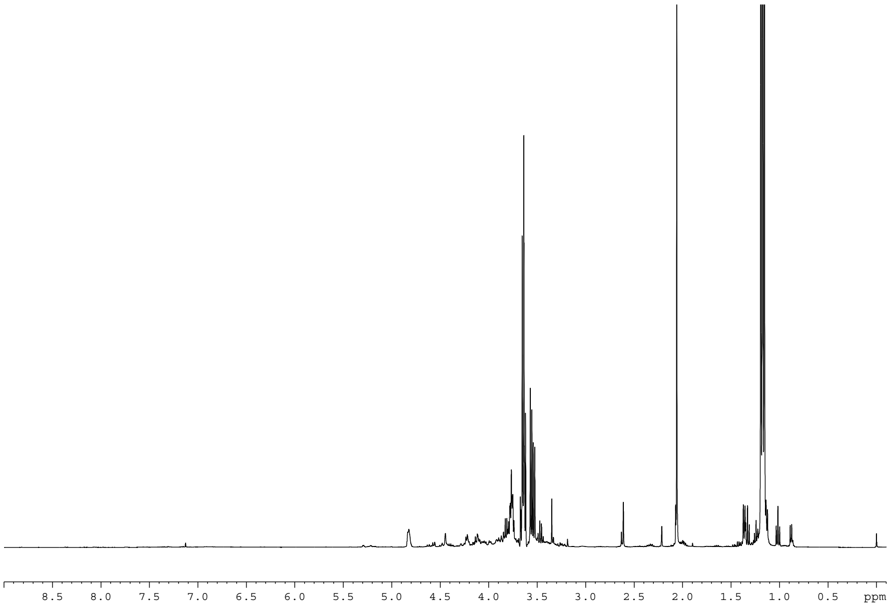


```
Current Data Parameters
NAME                 C06
EXPNO                 11
PROCNO                 1

F2 - Acquisition Parameters
Date_           20180316
Time               12.18
INSTRUM            spect
PROBHD   5 mm PABBI 1H/
PULPROG  noesygpps1d.comp1
TD                 65536
SOLVENT              D2O
NS                    32
DS                     4
SWH             8223.685 Hz
FIDRES          0.125483 Hz
AQ             3.9845889 sec
RG                    16
DW                60.800 usec
DE                 20.88 usec
TE                 300.0 K
D8            0.01000000 sec
D12           0.00002000 sec
D16           0.00010000 sec
L6                     4
TD0                    1

======== CHANNEL f1 ========
NUC1                  1H
P0                  8.94 usec
P1                  8.94 usec
P18           1000000.00 usec
PL1                -1.70 dB
PL9                59.27 dB
PL1W         12.92942619 W
PL9W          0.00001034 W
SFO1         400.1318819 MHz
SP6                41.00 dB
SPNAM[6]       primitivo
SPOAL6             0.500
SPOFFS6                0 Hz

====== GRADIENT CHANNEL =====
GPNAM[1]      SMSQ10.100
GPNAM[2]      SMSQ10.100
GPZ1               50.00 %
GPZ2              -10.00 %
P16              1000.00 usec

F2 - Processing parameters
SI                131072
SF           400.1299461 MHz
WDW                   EM
SSB                    0
LB                  0.30 Hz
GB                     0
PC                  4.00
```

g)

Wine Screening: second experiment, i.e. NOESYGPPS1D

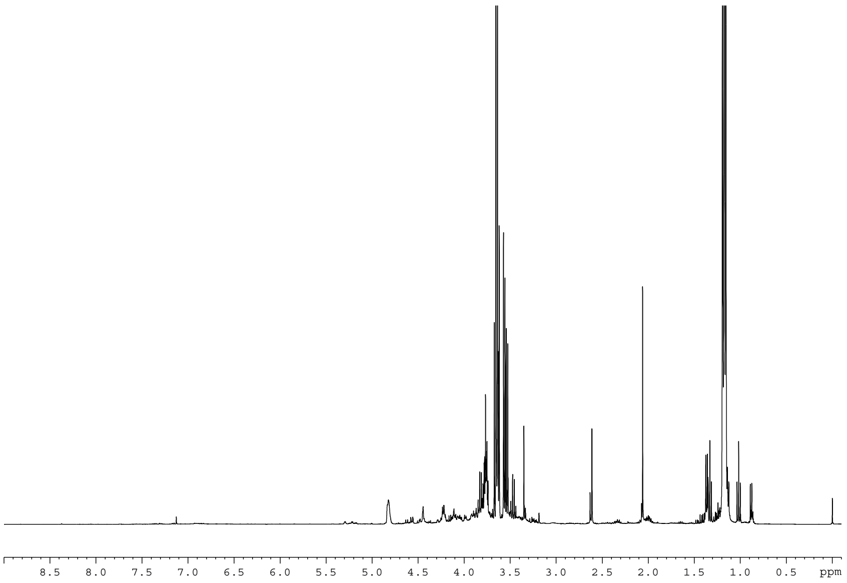


```
Current Data Parameters
NAME                 C11
EXPNO                 11
PROCNO                 1

F2 - Acquisition Parameters
Date_           20180316
Time               13.00
INSTRUM            spect
PROBHD   5 mm PABBI 1H/
PULPROG  noesygpps1d.comp1
TD                 65536
SOLVENT              D2O
NS                    32
DS                     4
SWH             8223.685 Hz
FIDRES          0.125483 Hz
AQ             3.9845889 sec
RG                    16
DW                60.800 usec
DE                 20.88 usec
TE                 300.0 K
D8            0.01000000 sec
D12           0.00002000 sec
D16           0.00010000 sec
L6                     4
TD0                    1

======== CHANNEL f1 ========
NUC1                  1H
P0                  8.94 usec
P1                  8.94 usec
P18           1000000.00 usec
PL1                -1.70 dB
PL9                59.27 dB
PL1W         12.92942619 W
PL9W          0.00001034 W
SFO1         400.1318819 MHz
SP6                41.00 dB
SPNAM[6]       primitivo
SPOAL6             0.500
SPOFFS6                0 Hz

====== GRADIENT CHANNEL =====
GPNAM[1]      SMSQ10.100
GPNAM[2]      SMSQ10.100
GPZ1               50.00 %
GPZ2              -10.00 %
P16              1000.00 usec

F2 - Processing parameters
SI                131072
SF           400.1299469 MHz
WDW                   EM
SSB                    0
LB                  0.30 Hz
GB                     0
PC                  4.00
```

h)

Wine Screening: second experiment, i.e. NOESYGPPS1D

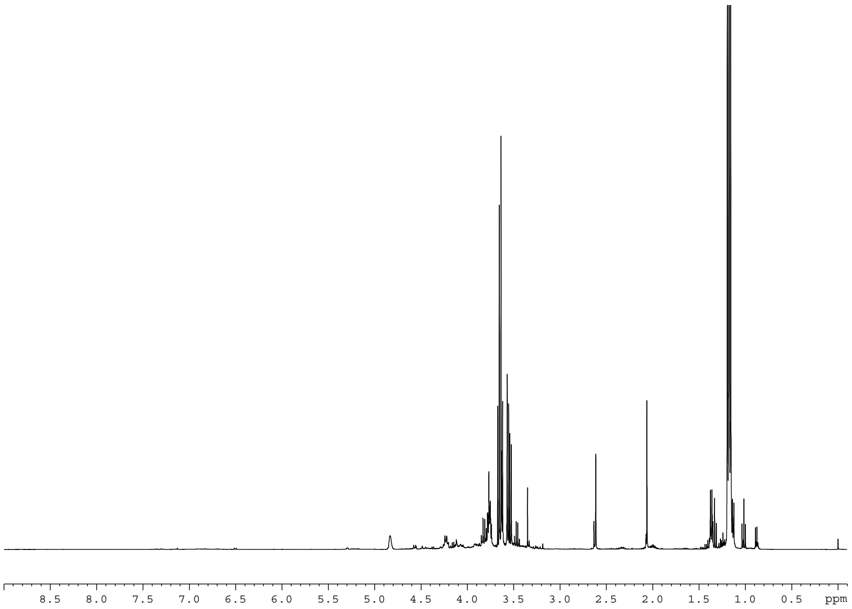


```
Current Data Parameters
NAME                 D07
EXPNO                 11
PROCNO                 1

F2 - Acquisition Parameters
Date_          20180316
Time              11.37
INSTRUM           spect
PROBHD   5 mm PABBI 1H/
PULPROG  noesygppsld.comp1
TD                65536
SOLVENT             D2O
NS                   32
DS                    4
SWH            8223.685 Hz
FIDRES         0.125483 Hz
AQ            3.9845889 sec
RG                   16
DW               60.800 usec
DE                20.88 usec
TE                300.0 K
D8           0.01000000 sec
D12          0.00002000 sec
D16          0.00010000 sec
L6                    4
TD0                   1

======== CHANNEL f1 ========
NUC1                 1H
P0                 8.94 usec
P1                 8.94 usec
P18          1000000.00 usec
PL1               -1.70 dB
PL9               59.27 dB
PL1W        12.92942619 W
PL9W         0.00001034 W
SFO1        400.1318819 MHz
SP6               41.00 dB
SPNAM[6]      primitivo
SPOAL6            0.500
SPOFFS6               0 Hz

====== GRADIENT CHANNEL =====
GPNAM[1]      SMSQ10.100
GPNAM[2]      SMSQ10.100
GPZ1              50.00 %
GPZ2             -10.00 %
P16             1000.00 usec

F2 - Processing parameters
SI               131072
SF          400.1299443 MHz
WDW                  EM
SSB                   0
LB                 0.30 Hz
GB                    0
PC                 4.00
```

i)

Wine Screening: second experiment, i.e. NOESYGPPS1D

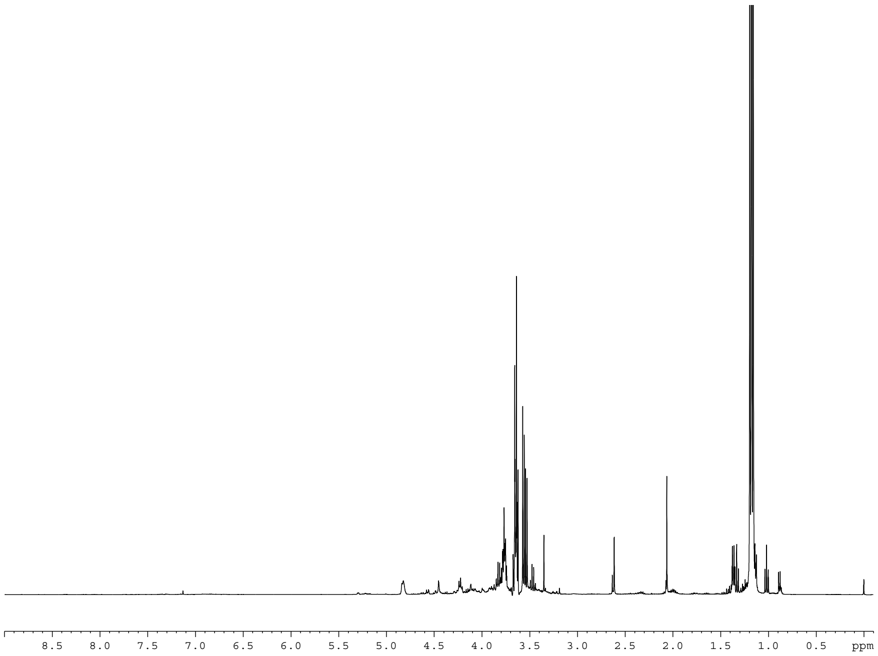


```
Current Data Parameters
NAME                 D09
EXPNO                 11
PROCNO                 1

F2 - Acquisition Parameters
Date_          20180316
Time              13.52
INSTRUM           spect
PROBHD   5 mm PABBI 1H/
PULPROG  noesygppsld.comp1
TD                65536
SOLVENT             D2O
NS                   32
DS                    4
SWH            8223.685 Hz
FIDRES         0.125483 Hz
AQ            3.9845889 sec
RG                   16
DW               60.800 usec
DE                20.88 usec
TE                300.0 K
D8           0.01000000 sec
D12          0.00002000 sec
D16          0.00010000 sec
L6                    4
TD0                   1

======== CHANNEL f1 ========
NUC1                 1H
P0                 8.94 usec
P1                 8.94 usec
P18          1000000.00 usec
PL1               -1.70 dB
PL9               59.27 dB
PL1W        12.92942619 W
PL9W         0.00001034 W
SFO1        400.1318819 MHz
SP6               41.00 dB
SPNAM[6]      primitivo
SPOAL6            0.500
SPOFFS6               0 Hz

====== GRADIENT CHANNEL =====
GPNAM[1]      SMSQ10.100
GPNAM[2]      SMSQ10.100
GPZ1              50.00 %
GPZ2             -10.00 %
P16             1000.00 usec

F2 - Processing parameters
SI               131072
SF          400.1299464 MHz
WDW                  EM
SSB                   0
LB                 0.30 Hz
GB                    0
PC                 4.00
```

j)

Wine Screening: second experiment, i.e. NOESYGPPS1D

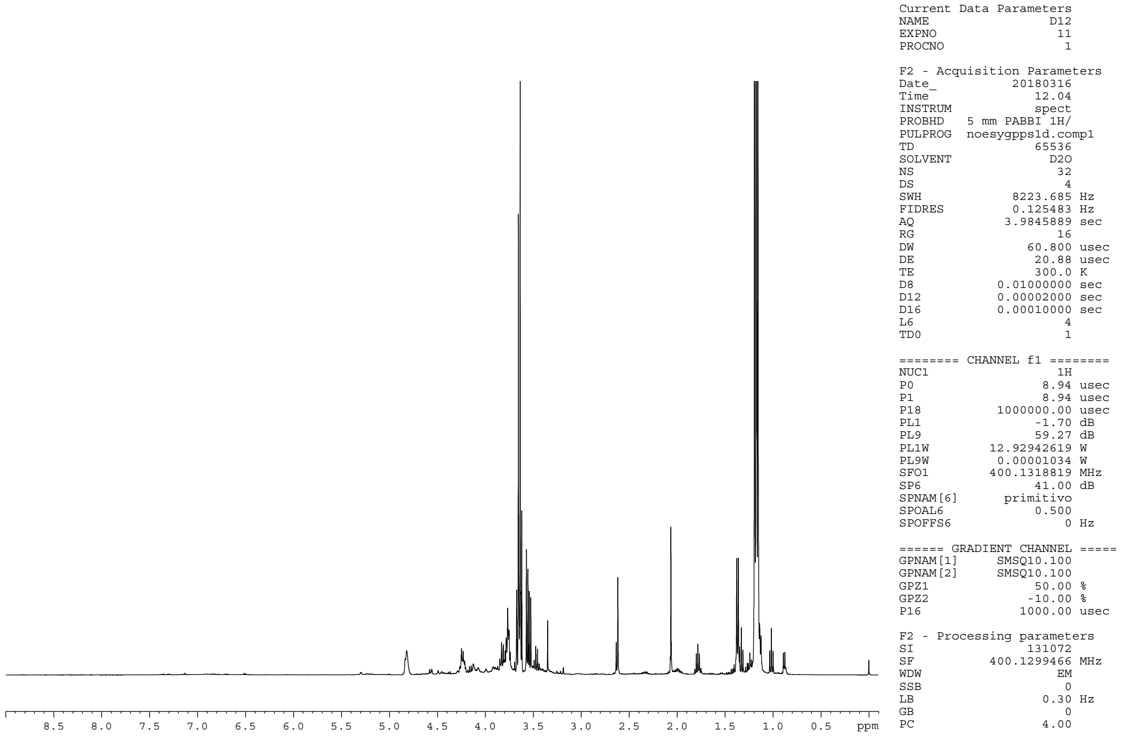

k)

Wine Screening: second experiment, i.e. NOESYGPPS1D

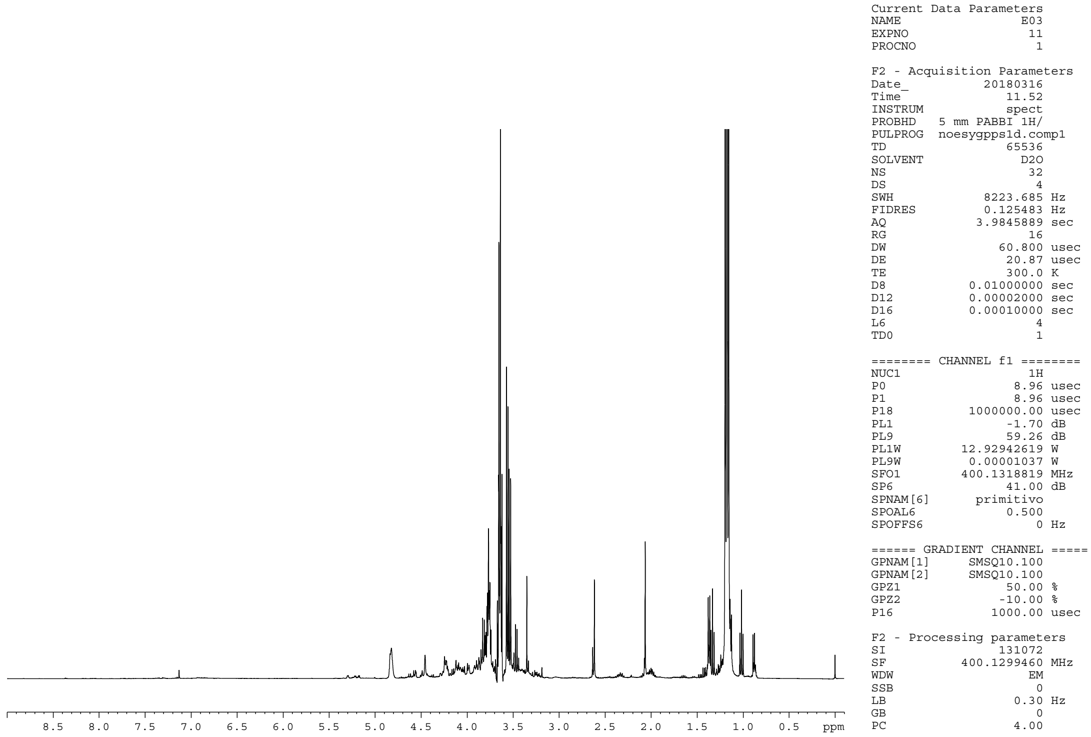

l)

Wine Screening: second experiment, i.e. NOESYGPPS1D

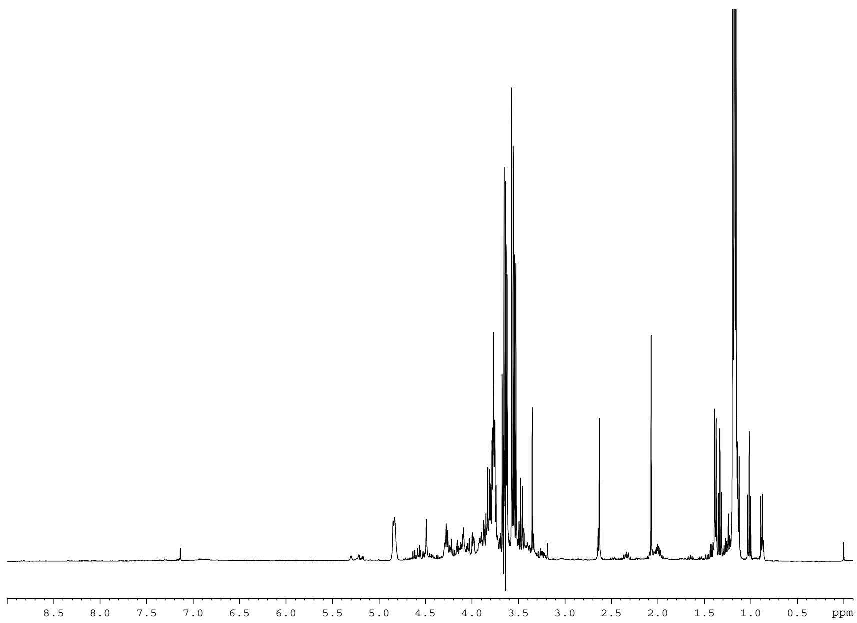

Current Data Parameters	
NAME	E04
EXPNO	11
PROCNO	1
**F2 - Acquisition Parameters**	
Date_	20180604
Time	14.45
INSTRUM	spect
PROBHD	5 mm PABBI 1H/
PULPROG	noesygppsld.comp1
TD	65536
SOLVENT	D2O
NS	32
DS	4
SWH	8223.685 Hz
FIDRES	0.125483 Hz
AQ	3.9845889 sec
RG	16
DW	60.800 usec
DE	20.90 usec
TE	300.0 K
D8	0.01000000 sec
D12	0.00002000 sec
D16	0.00010000 sec
L6	4
TD0	1
**======== CHANNEL f1 ========**	
NUC1	1H
P0	8.91 usec
P1	8.91 usec
P18	1000000.00 usec
PL1	-1.70 dB
PL9	59.30 dB
PL1W	12.92942619 W
PL9W	0.00001027 W
SFO1	400.1318819 MHz
SP6	41.00 dB
SPNAM[6]	primitivo
SPOAL6	0.500
SPOFFS6	0 Hz
**====== GRADIENT CHANNEL =====**	
GPNAM[1]	SMSQ10.100
GPNAM[2]	SMSQ10.100
GPZ1	50.00 %
GPZ2	-10.00 %
P16	1000.00 usec
**F2 - Processing parameters**	
SI	131072
SF	400.1299427 MHz
WDW	EM
SSB	0
LB	0.30 Hz
GB	0
PC	4.00

m)

Wine Screening: second experiment, i.e. NOESYGPPS1D

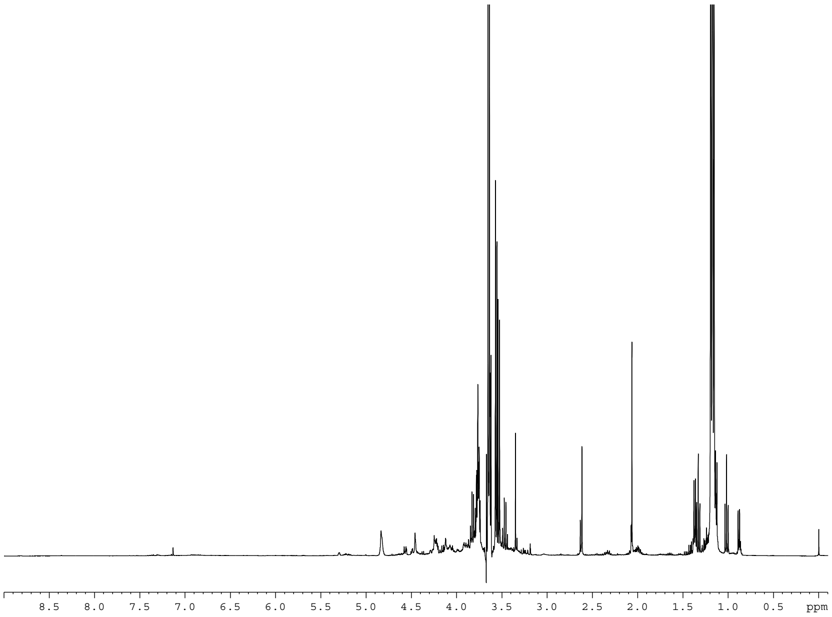

Current Data Parameters	
NAME	E10
EXPNO	11
PROCNO	1
**F2 - Acquisition Parameters**	
Date_	20180316
Time	12.41
INSTRUM	spect
PROBHD	5 mm PABBI 1H/
PULPROG	noesygppsld.comp1
TD	65536
SOLVENT	D2O
NS	32
DS	4
SWH	8223.685 Hz
FIDRES	0.125483 Hz
AQ	3.9845889 sec
RG	16
DW	60.800 usec
DE	20.88 usec
TE	300.0 K
D8	0.01000000 sec
D12	0.00002000 sec
D16	0.00010000 sec
L6	4
TD0	1
**======== CHANNEL f1 ========**	
NUC1	1H
P0	8.94 usec
P1	8.94 usec
P18	1000000.00 usec
PL1	-1.70 dB
PL9	59.27 dB
PL1W	12.92942619 W
PL9W	0.00001034 W
SFO1	400.1318819 MHz
SP6	41.00 dB
SPNAM[6]	primitivo
SPOAL6	0.500
SPOFFS6	0 Hz
**====== GRADIENT CHANNEL =====**	
GPNAM[1]	SMSQ10.100
GPNAM[2]	SMSQ10.100
GPZ1	50.00 %
GPZ2	-10.00 %
P16	1000.00 usec
**F2 - Processing parameters**	
SI	131072
SF	400.1299455 MHz
WDW	EM
SSB	0
LB	0.30 Hz
GB	0
PC	4.00

n)

Figure S2. ^{1}H NMR spectrum (noesygppsld.compl pulse program) of **a) b) c)** control; **d) e) f)** Primitivo fresh grape pomace; **g) h)** Primitivo stabilized grape pomace; **i) j) k)** Aglianico fresh grape pomace; **l) m) n)** Activated carbon treated wine samples

Table S1. Mean and standard deviation of total polyphenols (gallic acid content) and anthocyanins (malvin chloride) of wine samples (mg/L).

Wine	Polyphenols	Antocyanins
Control	3253.33±46.19	147.00±7.00
Repassage over fresh pomace of Primitivo	3006.67±255.80	115.33±12.74
Repassage over stabilized pomace of Primitivo	2923.33±134.29	128±8.19
Repassage over fresh pomace of Aglianico	2923.33±70.95	143.00±8.66
Activated carbon	2850.00±36.06	115.00±5.51

Table S2. ^{1}H-NMR signals (chemical shifts) of assigned metabolites in wine spectra

Compounds	Chemical Shifts (δH)
Isobutanol	0.88
Isopentanol	0.89; 1.64
2,3-Butanediol	1.14 (d) (J = 6.5 Hz)
Ethanol	1.18; 3.65
Lactate	1.37; 4.22
Alanine	3.86-3.79 , 1.47
Proline	2.37-2.28, 2.04-1.92
Acetate	2.05
Ethyl Acetate	2.07
Succinic Acid	2.62
GABA	3.00, 1.90
Methanol	3.35
Glycerol	3.59-3.50; 3.79-3.73
Tartaric Acid	4.47
β-Glucose	4.65
α -Xylose	5.17
α-Glucose	5.22
β-Arabinose	5.25
Galacturonic Acid	5.30
Sucrose	5.43
Caffeic Acid	6.43; 7.68
Tyrosol	6.85; 7.16
Tyrosine	6.93; 7.13
Gallic Acid	7.14
Formate	8.46
Trigonelline	9.10; 8.82;

Table S3. Performance and validation results to assess accuracy and robustness of PLS-DA model (Figure 4) for discrimination of untreated and treated wine samples, focusing aromatic spectral regions

Model parameters	PLS-DA
No of observations	14
R^2X	0.92
R^2Y	0.85
Q^2	0.568
NC[a]	5
Permutation tests (R^2; Q^2)	0.479 : -0.4.91
ROC	1
Fisher's probability	$1.8*10^{-6}$
CCR (%)[c]	100
